# Population Genetics and Trajectory Simulation Reveals the Invasion Process of the Fall Armyworm (*Spodoptera frugiperda*) in the Eastern Hemisphere

**DOI:** 10.1111/eva.70086

**Published:** 2025-02-24

**Authors:** Pengfei Fu, Xijie Li, Zhongxiang Sun, Yaping Chen, Zhihui Lu, Caihong Tian, Gao Hu, Furong Gui

**Affiliations:** ^1^ State Key Laboratory of Conservation and Utilization of Biological Resources of Yunnan College of Plant Protection, Yunnan Agricultural University Kunming China; ^2^ Department of Entomology College of Plant Protection, Nanjing Agricultural University Nanjing China; ^3^ Department of Agronomy and Horticulture Jiangsu Vocational College of Agriculture and Forestry Jurong China; ^4^ Institute of Plant Protection, Henan Academy of Agricultural Sciences Zhengzhou Henan Province China

**Keywords:** Eastern Hemisphere, fall armyworm, genetic evolution, invasion process, migration trajectory

## Abstract

As a migratory agricultural pest, the fall armyworm has been in the spotlight since it invaded Africa in 2016. Invasive populations have now colonized much of the Eastern Hemisphere, causing severe damage to a wide range of crops. However, there is still disagreement internationally on the origin and mode of invasion of fall armyworm populations, especially from the Americas to China. In this study, we provided an in‐depth insight into the invasion of the fall armyworm in the Eastern hemisphere based on genome‐wide data from 124 fall armyworm individuals collected from 14 sites across three continents and trajectory simulation. First, based on 770,423 high‐quality SNPs, the PCA and ADMIXTURE analyses clearly distinguished the geographical populations of the Eastern and Western hemispheres. Second, the genetic diversity results revealed that the invasive populations exhibited higher heterozygosity than the native populations. Third, the results of integrated individual assignment tests and migration path simulations showed that the W1 (Florida, Texas, and Puerto Rico) population may be the potential source of the invasive populations in Africa, and a low possibility of trans‐sea migration between the Americas and Africa suggested that fall armyworms may have spread through trade in goods. Fourth, our results indicate that the Indian population is a genetically admixed group derived from the E1 (Benin, Ethiopia, and South Africa) population, which subsequently migrated to the Indo‐China Peninsula through natural trans‐sea dispersal and to Yunnan via Myanmar. These findings not only provide new insights into the invasion of the fall armyworm in the Eastern Hemisphere but also present a method to improve the prediction accuracy of migratory pests.

## 1 Introduction

The fall armyworm ( *Spodoptera frugiperda* J.E.Smith) is a highly polyphagous pest found throughout the tropical and subtropical regions of the Americas, which possesses strong migratory, adaptability, and destructive characteristics (Day et al. 2017; Westbrook and Sparks 1986). In 2016, it invaded Africa and subsequently spread to Asia, posing a significant threat to global food security (Ganiger et al. 2018; Goergen et al. 2016; IPPC 2018; Jing et al. 2020). Therefore, studying its worldwide migratory patterns will enhance understanding of the mechanisms underlying its dispersion and the resulting threats. However, there is still disagreement internationally on the origin and mode of invasion of fall armyworm populations, especially from the Americas to China.

The fall armyworm's spread from the Americas to China required three major cross‐border migrations. The first was the migration from America to Africa. However, different hypotheses have been given based on various research methods. For example, it has been suggested that the African armyworm originated from Florida and the Caribbean region, with the possibility of multiple introductions (Nagoshi et al. 2018; Schlum et al. 2021). Another suggestion has been that populations from Mississippi in the United States and Brazil could be the source of the African fall armyworm invasion (Tay et al. 2022). A recent study has also suggested that the invasive population is more closely related to the North and Central American populations and therefore is less likely to originate from South America (Zhang et al. 2023). Meanwhile, the prevailing trade winds are from Africa to the Americas, making unaided dispersal by adult flight a very unlikely pathway of entry in this case. It is now generally accepted that the possible route of entry to Africa is by transportation of adults or egg masses on direct commercial flights between the Americas and West Africa (Cock et al. 2017). While most studies suggested that Florida and the Greater Antilles are the likely sources of at least a subset of the African infestation, there remains ongoing controversy regarding the second origin.

The second cross‐border migration of the fall armyworm's spread from the Americas to China is the migration from Africa to India. The Indian population has been reported to show genetic homogeneity with the South African and East African populations (Nagoshi et al. 2019).

The third cross‐border migrations of the fall armyworm's spread from the Americas to China is the migration from India to the Indo‐China Peninsula. However, there is still controversy about the origin of the Chinese fall armyworm. Comparison of the mitochondrial genome (COI) and nuclear genome (TPI) markers revealed the Southeast Asian fall armyworm is closely related to African and Indian populations, suggesting a common origin (Nagoshi et al. 2020). Additionally, it is consistent with the conclusion that the genetic structure of the Chinese population is more similar to that of the African population (Gui et al. 2022). However, a genome‐wide analysis of different characteristics between the Myanmar and Chinese fall armyworm populations, which identified 870 SNPs, did not support the hypothesis of “spread of African origin” or “spread of Myanmar populations into China” (Rane et al. 2023).

Genetic methods can be applied to study the migration of the fall armyworm. For instance, the COI and TPI markers have been widely applied to study the fall armyworm migration (Martinelli et al. 2006; Zhang et al. 2020). However, the fall armyworm is a significant transboundary migratory pest with an extraordinary ability for migration and rapid genetic exchange. Traditional methods of analysis based on microsatellite markers, such as COI and TPI genes, often fail to accurately reflect its complex population structure and migration dynamics in the invaded region (Hurst and Jiggins 2005). Whole‐genome analysis offers a higher resolution and is capable of effectively investigating the population structure of the fall armyworm across different regions. However, few studies have focused on the whole genome in studies on the fall armyworm migration (Ganiger et al. 2018; Goergen et al. 2016; IPPC 2018; Nagoshi et al. 2007, 2010, 2012, 2015).

In this study, we provide new insights into the invasion of the fall armyworm in the Eastern Hemisphere, based on genome‐wide data collected from 124 fall armyworm individuals collected from 14 sites across three continents. This included data from the native representative populations (Americas) and the invasive representative populations (Africa, India, Southeast Asia, and China). We focused specifically on the Myanmar, Laos, and Vietnam populations to capture an exhaustive representation of the species' genetic heterogeneity. This strategy bolsters the credibility of our analyses of the transcontinental migration routes of the fall armyworm from Africa to Asia. In addition, we also verified the results by simulating its migratory trajectories. Here, we reassess the global migration patterns of the fall armyworm by simulating its migratory trajectories on a global scale, utilizing genome‐wide data to improve the accuracy of invasion predictions.

## 2 Materials and Methods

### 2.1 Sample Collection

In this study, we used re‐sequencing data of fall armyworm samples collected in Kenya, Ethiopia, South Africa, and Yunnan province, China (39 individuals) (CNSA: CNP0001020), which were generated from our previous studies (Gui et al. 2022). We also collected re‐sequencing data of fall armyworm samples collected from Katrina A. Schlum in Florida, Texas, Puerto Rico, and Brazil (26 individuals) (PRJNA640063) (Schlum et al. 2021); from Sudeeptha Yainna in Mexico, Benin, and India (30 individuals) (PRJNA639295) (Yainna et al. 2022); and from Rane Rahul in Myanmar, Laos, and Vietnam (29 individuals) (Rane et al. 2023). The total number of individuals used in this study was 124 (36 from native populations and 88 from invasive populations) (Table S1 and Figure 6). The re‐sequencing data cover a large proportion of the distribution of the fall armyworm, especially in Southeast Asia, which helped to explore its invasion in the Eastern Hemisphere.

### 2.2 SNP Genotyping and Filtering

The tool, fastp (v0.20.0), was employed to assess read quality, and the sliding window method was used to filter the raw data (Chen et al. 2018). Low‐quality reads with a Phred score < 20 were removed. Reads in which either of the paired ends had a length < 50 bp or the number of N bases was ≥ 5 were discarded. Meanwhile, adapter contamination at the 3′ end was eliminated.

The clean reads were aligned to the fall armyworm Genome (https://www.ncbi.nlm.nih.gov/datasets/genome/GCF_011064685.2/) using the bwa mem program (0.7.12‐r1039); the comparison parameters were based on the default parameters of bwa mem (Li and Durbin 2009; Xiao et al. 2020). Picard (v1.107) (http://www.psc.edu/index.php/user‐resources/software/picard) was used for sorting the Sam file, which was converted to the bam file. The “FixMateInformation” command was used to ensure consistency among all paired‐end reads. Duplicates were removed using the MarkDuplicates function in the Picard package. The IndelRealigner command in the GATK program (v3.8) was used to recompare reads near InDel to improve the accuracy of SNP Calling.

SNPs were identified using the GATK software (v3.8), which was used to generate a VCF file according to the following criteria: the RealignerTargetCreator command and IndelRealigner command in the GATK software package were used to recompare all reads near InDels (Zhu et al. 2015). The UnifiedGenotyper program was used to obtain the SNP site of the sample, with stand_call_conf set to 30 and stand_emit_conf set to 10. This resulted in a VCF file containing 28,496,237 SNPs, which were obtained using the filter parameters: (1) Fisher test of strand bias (FS) ≤ 60; (2) HaplotypeScore ≤ 13.0; (3) Mapping Quality (MQ) ≥ 40; (4) Quality Depth (QD) ≥ 2; (5) ReadPosRankSum ≥ −8.0; (6) MQRankSum > −12.5.

### 2.3 Genetic Diversity and Phylogenetic Tree

After applying the initial filtering criteria, a subset of high‐quality SNPs (770,423) was further filtered using PLINK (v1.9) with the parameters, reads depth ≥ 3; minor allele frequency ≥ 0.05; maximum missing rate ≤ 0.2, and used for distance tree construction and population structure analysis (Purcell et al. 2007).

For samples from multiple populations, observed (Hobs), expected (Hexp) heterozygosity, nucleotide diversity (PI), Wright's inbreeding coefficient (FIS), and FST were calculated for each population using the populations program in Stacks.

The maximum likelihood algorithm in the software FastTree (http://www.microbesonline.org/fasttree/) was employed to construct a phylogenetic tree using 124 individual fall armyworm samples, with three *Spodoptera litura* individuals used as outgroups (GenBank: SRR5132417, SRR5132423, SRR5132429) (Price et al. 2009). The reliability of the phylogenetic tree branches was validated using the bootstrap method with 1000 replications.

### 2.4 Population Structure

Principal component analysis was performed using the SNP data (excluding SNPs with MAF less than 0.05) using the GCTA software (Genome‐wide Complex Trait Analysis, version 1.94.1; https://yanglab.westlake.edu.cn/software/gcta/), which enables large‐scale SNP data analysis for investigating the genetic architecture of complex traits. This tool efficiently handles high‐dimensional genomic data and computes eigenvectors for PCA to cluster individuals into subgroups based on principal components (Yang et al. 2011). For population genetic structure analysis, the Admixture software (version 1.3.0; http://dalexander.github.io/admixture), a powerful tool for estimating individual ancestry proportions from SNP data, was employed. Admixture applies a maximum likelihood method to infer ancestral population structure and admixture proportions (Alexander et al. 2009). A mixed model approach was used for the analysis with *K* = 1–10, where K represents the number of ancestral populations. This range was chosen based on standard practices in population structure analyses to ensure the model's stability and explore biological plausibility. The cross‐validation (CV) procedure in admixture was applied to determine the optimal *K* value by minimizing the CV error, thereby balancing model fit and biological interpretability. Default software parameters were used for all analyses unless specified otherwise. CV error values across different *K* values were examined, and the *K* value closest to the true ancestral population was selected.

### 2.5 Assignment Tests

Assignment tests were performed in assignPOP (v.1.1.7) (Chen et al. 2017, 2021) to evaluate the ability of genetic data to differentiate native (W1, W2, and W3) and invasive (E1 and E2) populations. The W1, W2, and W3 (native populations) and E1 and E2 (invasive populations) source groups were trained using the support vector machine algorithm to build predictive models. For training, we used 50%, 70%, and 90% random individuals from each group, and loci with the highest 60%, 80%, and 100% FST values. Using the default variance threshold, *p* = 0.95, low variance sites in the dataset were removed for further analysis. Monte Carlo cross‐validation was performed by resampling each training set combination 1000 times. We hypothesized that the support vector machine algorithm would successfully assign individuals from invasive populations to their most likely source groups based on their genetic composition. This approach aimed to test the hypothesis that invasive populations (e.g., those in Africa and India) share genetic similarities with their presumed source groups and to trace the genetic structure of population migrations. These assignment tests are essential for validating the hypothesis that invasive populations exhibit genetic overlap with native source groups, providing evidence for population connectivity and the potential routes of invasion.

### 2.6 Simulation of Migratory Trajectory

We simulated the potential trans‐regional migration of fall armyworm without the involvement of human activities. In order to improve the accuracy of the simulation, we applied a new trajectory analysis model and trajectory calculation based on high spatiotemporal resolution weather conditions simulated by the Weather Research and Forecasting (WRF) model (Chapman et al. 2010, 2012; Li et al. 2019). The program for calculating trajectories was designed in FORTRAN and run under CentOS 7.4 on a server platform (IBM system x3500 M4) (Hu et al. 2013; Wu et al. 2018). The starting point of fall armyworm flight was set for America (81W, 27N; 66W, 18N; 43W, 21N), East Africa (50E, 10N), India (80E, 16N; 77E, 13N; 80E, 13N; 77E, 16N; 83E, 19N; 80E, 19N), and Myanmar (96E, 18N; 99E, 21N; 96E, 24N), the departure time was set at 19:00 local time, and the stopping time was 5:00 the next day. The fall armyworm was set to fly downwind at a speed of 3 m/s. The single flight time after entering the sea was extended, and the flight was not actively stopped. In the simulation of trans‐sea migration flight, eight different altitudes were used for trajectory calculation. These were 500, 750, 1000, 1250, 1500, 1750, 2000, and 2250 m above sea level. An altitude of 1500 m above the underlying surface was used in the case analysis and the Myanmar to China migration case.

## 3 Results

### 3.1 SNP Filtering

We analyzed genome‐wide data collected from 36 fall armyworm individuals in the Western Hemisphere (native populations) and 88 fall armyworm individuals in the Eastern Hemisphere (invasive populations) (Table S1 and Figure 6). In total, we individually aligned the clean reads of 124 samples to the reference genome, ZJU_Sfru_1.1 (https://www.ncbi.nlm.nih.gov/datasets/genome/GCF_011064685.2/). The mapping rate of the alignment ranged between 61.18% and 98.4%. The read depth coverage across 124 samples ranged from 3.4× to 88.17×, with an average depth of 21.39× (Tables S2 and S3). A dataset of 28,496,237 SNPs was obtained after preliminary filtering. Subsequently, a total of 770,423 high‐quality SNPs was retained for further analyses.

### 3.2 Population Structure and Phylogenetic Tree

**FIGURE 1:**
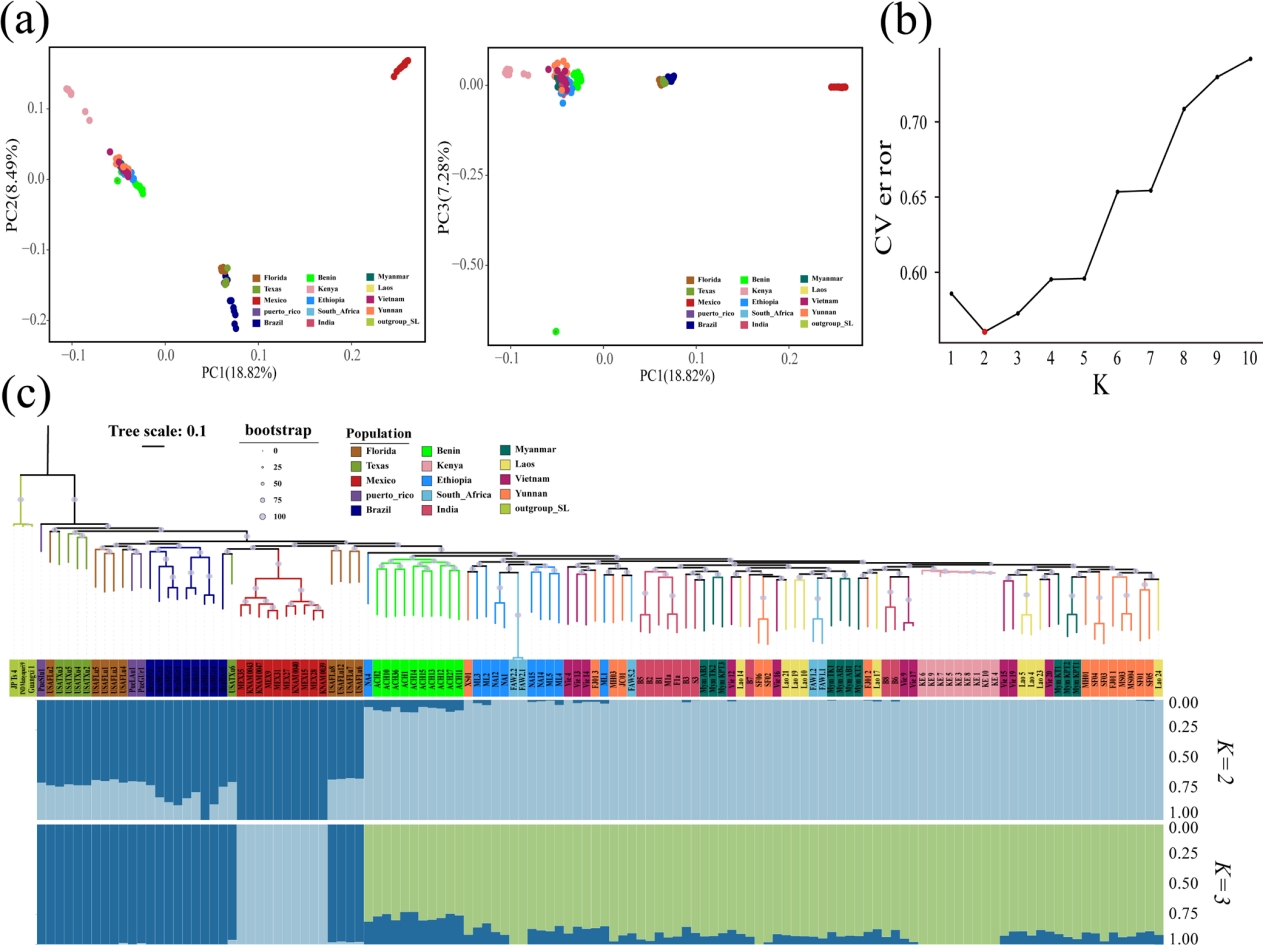
Population genetic structure analysis of the fall armyworm. (a) PCA plot of the first and second components and the first and third components. The horizontal and vertical coordinates represent the contributions of each principal component. Populations in different countries are color‐coded. (b) The value of *K* represents the assumed number of ancestral populations. The best‐fitting ADMIXTURE model is *K* = 2 and *K* = 3, suggested by the cross‐validation error. (c) Maximum likelihood algorithm clustering analysis with three *Spodoptera litura* used as the outgroup, and branch length information was retained. The dots of different sizes on the branches of the developmental tree represent Bootstrap values of different sizes. Population structure inferred by ADMIXTURE. The population structure at *K* = 2 and *K* = 3 for the best‐fitting ADMIXTURE models.

Principal component analysis (PCA), phylogeny tree, and ADMIXTURE analyses were performed to further explore the genetic structure of the fall armyworm samples. PCA analysis in the first three PCs explained 18.82%, 8.49%, and 7.28% of the total variation, which distinguished 124 fall armyworm individuals into three clusters. The Mexican population clustered as an independent group, and the remaining samples separated into two clusters in the Eastern and Western hemispheres (Figure 1a). Some individuals in the Eastern Hemisphere population, such as the Kenyan population and some individuals in South Africa, deviated from the second and third principal components (Figure 1a).

A neighbor‐joining phylogeny tree was constructed with three *Spodoptera litura* individuals as an outgroup. The results of the phylogeny tree showed that, in the Western Hemisphere branch, the Florida population, Texas population from North America, and the Puerto Rican population from Central America clustered together (Figure 1c). The Brazil population from South America formed a cluster. The Mexico population from Central America also isolated in a single branch. In the Eastern Hemisphere branch, the Africa and Asia populations formed a large cluster nested within the native population (Figure 1c). The results also showed that the Kenyan population had a short branch length and distinct population differentiation characteristics, which may represent the relatively differentiated populations detected in the Eastern Hemisphere (Figure 1c).

Consistent with PCA and the phylogeny tree results, ADMIXTURE analysis clearly distinguished samples from the Eastern and Western hemispheres at *K* = 2 (Figure 1b). The Mexican population distinguished alone at *K* = 3 (Figure 1c). The remaining samples clearly distinguished into Eastern and Western hemisphere samples, where *K* represents the number of hypothetical ancestral populations (Figure 1c). The cross‐validation error indicated that the optimal ADMIXTURE model was *K* = 2, indicating that the PCA and ADMIXTURE analyses clearly distinguished the geographical populations of the Eastern and Western hemispheres but could not explain the genetic relationship among the individuals (Figure 1b,c).

The genetic structure of 124 fall armyworms from the Eastern and Western hemispheres was analyzed by Phylogenetic tree, PCA, and ADMIXTURE analysis. At least three relatively independent genetic clusters were identified from the Western Hemisphere: W1 (Florida, Texas, and Puerto Rico), W2 (Brazil), and W3 (Mexico) (Figure 1a). These clusters exhibited similar genetic backgrounds, with the W3 cluster likely having an earlier geographic origin (Figure 1c). At least two relatively independent genetic clusters were identified in the African population: E1 (Benin, Ethiopia and South Africa) and E2 (Kenya) (Figure 1a,c). The E1 cluster represented the early invasive population from the Western Hemisphere to the Eastern Hemisphere (Figure 1c). The E2 cluster represented a likely earlier invasive group in Africa (Figure 1a,c). Other E3 (Indian), E4 (Southeast Asian), and E5 (Yunnan) populations represented the rapid migration and spread of the invasive populations throughout Asia (Figure 1c).

### 3.3 Genomic Differentiation

**FIGURE 2:**
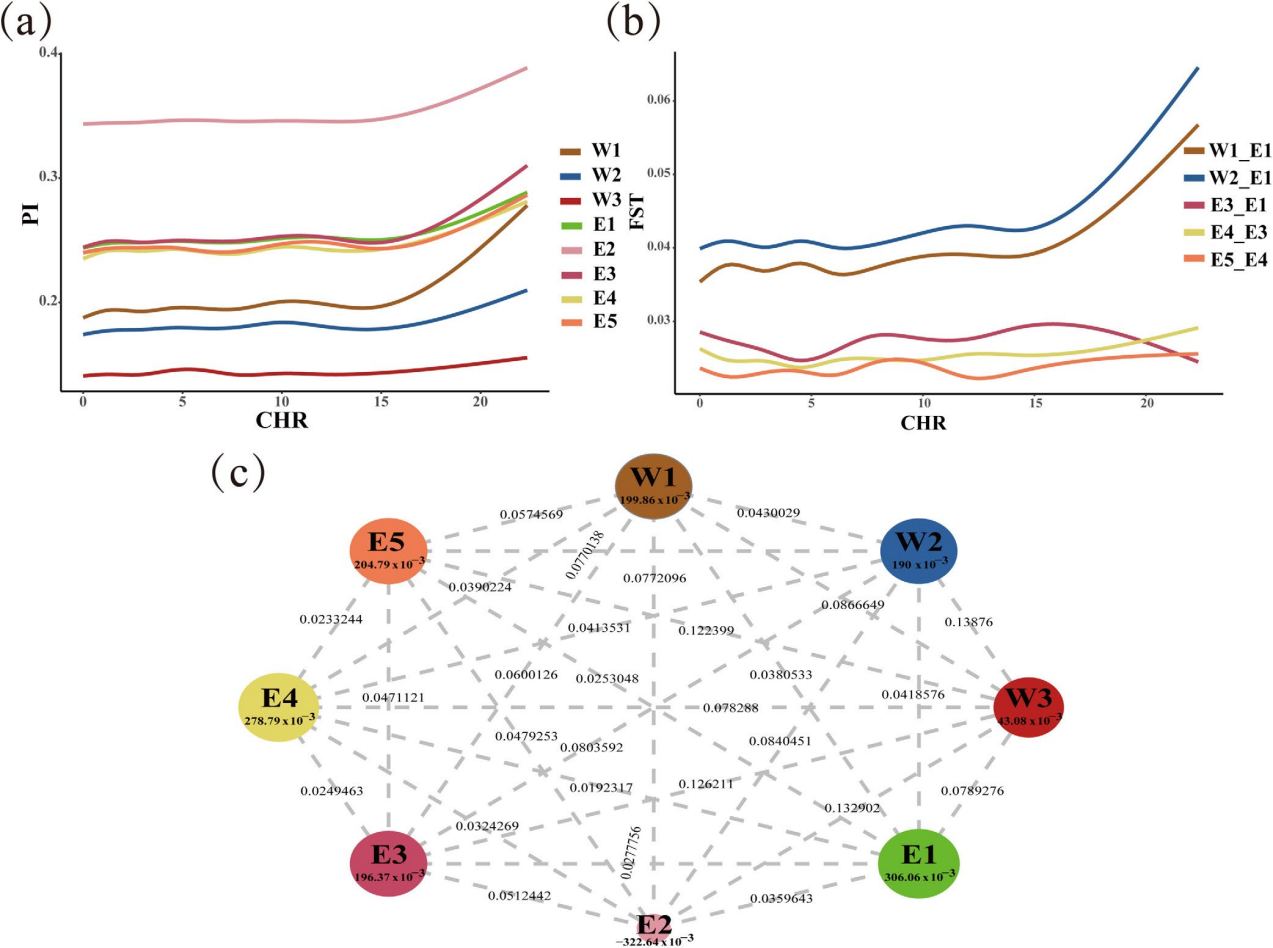
Nucleotide diversity (PI), inbreeding coefficient (FST) and inbreeding coefficient (FIS) of fall armyworm. (a) PI, the genome‐wide distribution of nucleotide diversity (PI) values of eight clusters (W1–W3, E1–E5); CHR, chromosome. (b) FST, The genome‐wide distribution of FST values of the W1–E1, W2–E1, E3–E1, E4–E3, and E5–E4; CHR, chromosome. (c) The FIS and FST of the eight genetic clusters. The size of the circle indicates the FIS value, with larger circles indicating a higher proportion of inbreeding within the population, and smaller circles indicating a higher proportion of outbreeding within the population. The lines between the circles and the accompanying numerical values represent the FST values between two populations. A larger FST value indicates greater genetic differentiation between populations, while a smaller FST value indicates closer genetic relatedness.

The genetic diversity of the 124 fall armyworms was analyzed based on whole‐genome data. The average number of individuals per locus (Num Indv) was determined to be 12.996, ranging from 6.385 to 24.912 (Table 1). Observed heterozygosity (OHe) was lower than expected heterozygosity (EHe) in all populations (except E2) (Table 1). The OHe of E2 was higher than the EHe, indicating that the E2 population may have a selective advantage of heterozygosity (Table 1). Average nucleotide diversity (PI) estimated for each population ranged from 0.143 to 0.346 (Table 1). In addition, the results also revealed that the invasive samples from the E2 cluster exhibited the highest average heterozygosity followed by those from the E1, E3, E4, and E5 clusters. The W1, W2, and W3 clusters from the Western Hemisphere exhibited lower PI values and showed a different nucleotide diversity than those from the Eastern Hemisphere, suggesting that the fall armyworm had experienced a rapid increase in nucleotide diversity since the Western hemisphere invasion (Table 1 and Figure 2a). In addition to the Kenyan population, all populations deviated from Hardy–Weinberg equilibrium with an apparent inbreeding coefficient (FIS) ranging from 0.043 to 0.306 (Table 1 and Figure 2c).

**TABLE 1:** Population genetic diversity of fall armyworm.

Cluster	Num_Indv	OHe	OHo	EHe	EHo	PI	FIS
W1	14.52303	0.13454	0.86546	0.18957	0.81043	0.19645	0.19986
W2	6.38469	0.10126	0.89874	0.16458	0.83542	0.17959	0.19000
W3	8.80933	0.12427	0.87573	0.13466	0.86534	0.14304	0.04308
E1	22.87936	0.15868	0.84132	0.24407	0.75593	0.24955	0.30606
E2	8.99595	0.50033	0.49967	0.32711	0.67289	0.34636	−0.32264
E3	8.66512	0.16862	0.83138	0.22773	0.77227	0.24198	0.19637
E4	24.91157	0.1692	0.8308	0.24511	0.75489	0.25017	0.27879
E5	8.80566	0.1686	0.8314	0.23005	0.76995	0.24416	0.20479

*Note:* All populations deviated the from Hardy–Weinberg equilibrium (except Kenya).

Abbreviations: EHe, the average expected heterozygosity of all loci in the population; EHo, the average expected homozygosity of all sites in the population; FIS, inbreeding coefficient; Num Indv, the average number of individuals at each site in the population; OHe, the average observed heterozygosity of all sites in the population; OHo, the average observed homozygosity of all sites in the population; PI, the average nucleotide diversity of all sites in the population.

We also assessed the genetic differentiation (FST) between the eight genetic clusters and found that the native clusters (W1 and W2) and invasive clusters (E1 and E2) exhibited lower FST value compared to the W3 cluster, suggesting a closer genetic relationship among the E1, E2, W1, and W2 clusters (Table S4 and Figure 2b,c). Notably, in the Eastern hemisphere, the FST values for E3 and E1, E4 and E3, and E5 and E4 showed a decreasing trend from high to low, which is consistent with the view of “spread of African origin” (Table S4 and Figure 2b,c).

Overall, these results highlight the complexity of genetic diversity and population distribution in fall armyworm. Our analysis supports the view of a migration from the Western Hemisphere to the Eastern Hemisphere and “spread of African origin.”

### 3.4 Migration From Americas to Africa

The fall armyworm's spread from the Americas to China required three major cross‐border migrations. The first was the migration from America to Africa, the second was the migration from Africa to India, and the third was the migration from India to the Indo‐China Peninsula.

**FIGURE 3:**
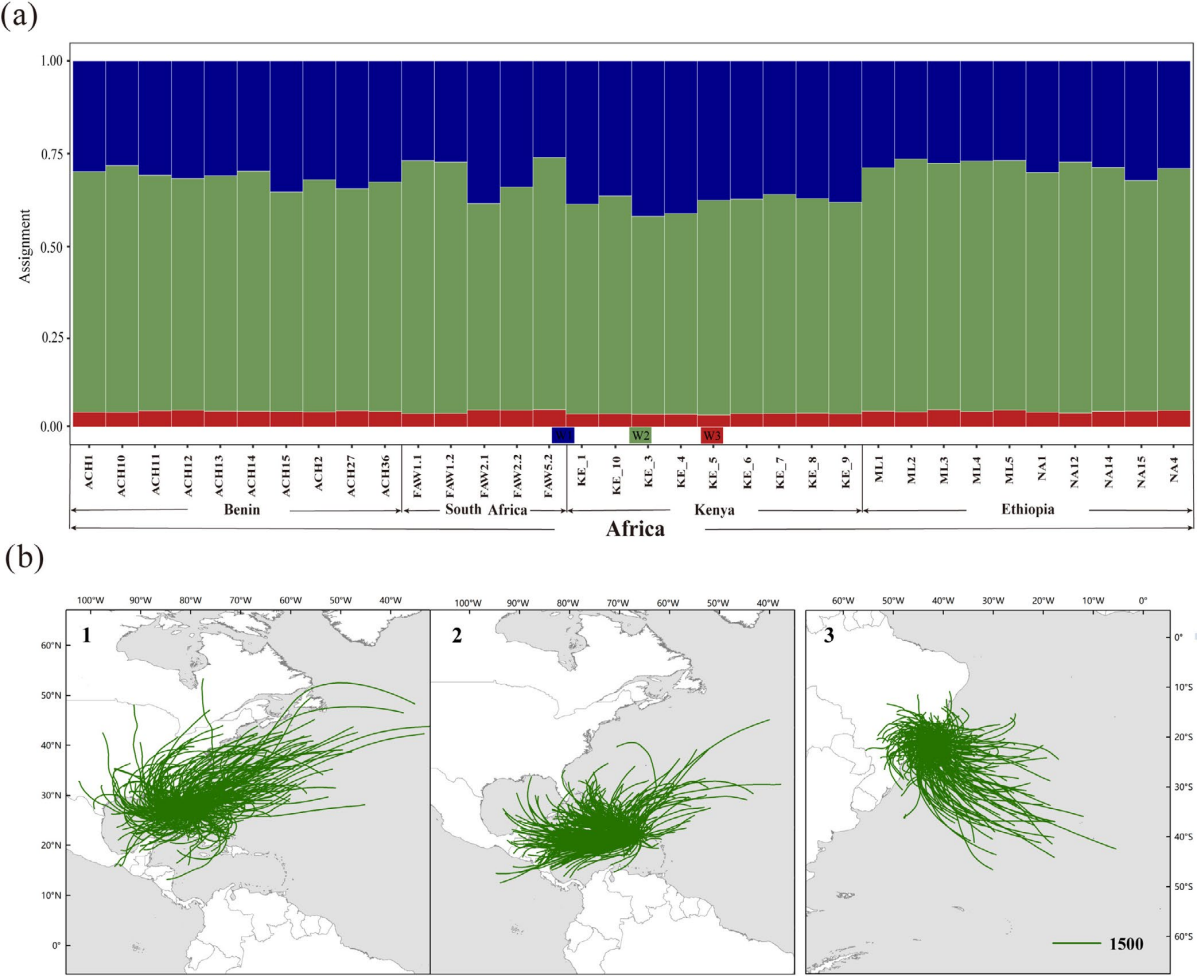
The assignment and simulated trajectories of the trans‐sea migration of fall armyworm from the Americas to Africa. (a) Assignment proportions of individuals were estimated using the R package assignPOP. Each individual was assigned to three source groups: W1, W2, and W3. (b) Simulated trajectories of trans‐sea migration of fall armyworm from the Americas to Africa between January 2015 and February 2016. (b1) Simulated trajectory of fall armyworm migration from North America to Africa. (b2) Simulated trajectory of fall armyworm migration from Central America to Africa. (b3) Simulated trajectory of fall armyworm migration from South America to Africa.

We conducted individual assignment tests and migration path simulations for the fall armyworm's migration from the Americas to Africa. In the assignment test, three source groups: W1, W2, and W3 were trained using the support vector machine algorithm to build predictive models. The results showed that 100% of African individuals were assigned to W1 (Table S5 and Figure 3a). To explore the possibility of natural trans‐sea migration of the fall armyworm between the Americas and Africa, we mapped the wind field between the two continents during 2015 and simulated the migration of the fall armyworm between January 2015 and February 2016 (Figure S1 and Figure 3b). The results showed that the prevailing wind direction is from Africa to the Americas, and the results of the trajectory simulation supported the view that it is impossible for natural trans‐sea migration from the Americas to Africa (Cock et al. 2017).

### 3.5 Spread From Africa to India

**FIGURE 4:**
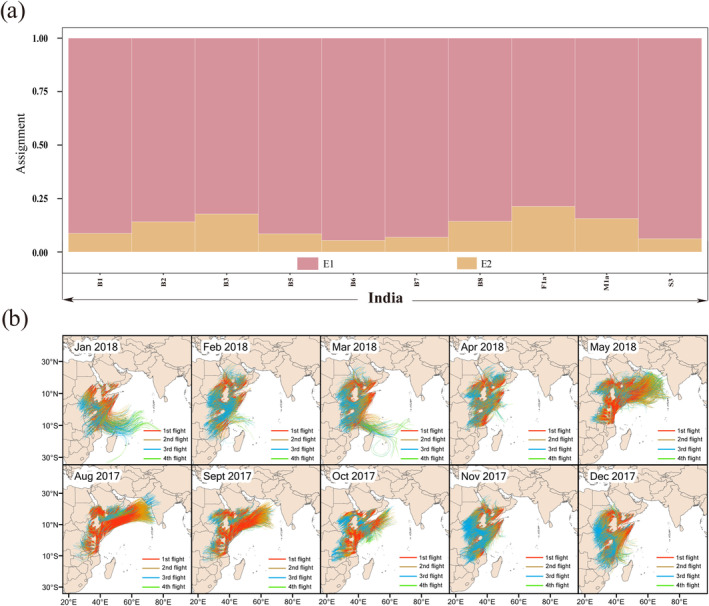
The assignment and simulated trajectories of the trans‐sea migration of fall armyworm from Africa to India. (a) Assignment proportions of individuals were estimated using the R package assignPOP. Each individual was assigned to two source groups: E1 and E2. (b) The simulated trajectories of the trans‐sea migration of fall armyworm from Africa to India in August 2017 to May 2018.

In the assignment test, two source groups: E1 and E2 were trained using the support vector machine algorithm to build predictive models. The results showed that 100% of Indian individuals were assigned to E1 (Table S6 and Figure 4a). In addition, analysis of the background weather during the invasion from Africa to Asia indicated a possibility of rapid trans‐regional spread through trans‐sea migration flight of the fall armyworm from Africa. The results showed that natural migration across the sea to India from September 2017 to May 2018 was impossible. However, this was possible in August 2017 (Figure 4b). Therefore, it is speculated that the fall armyworms discovered in India were descendants of the migrating population from August 2017.

### 3.6 From India to the Indo‐China Peninsula

**FIGURE 5:**
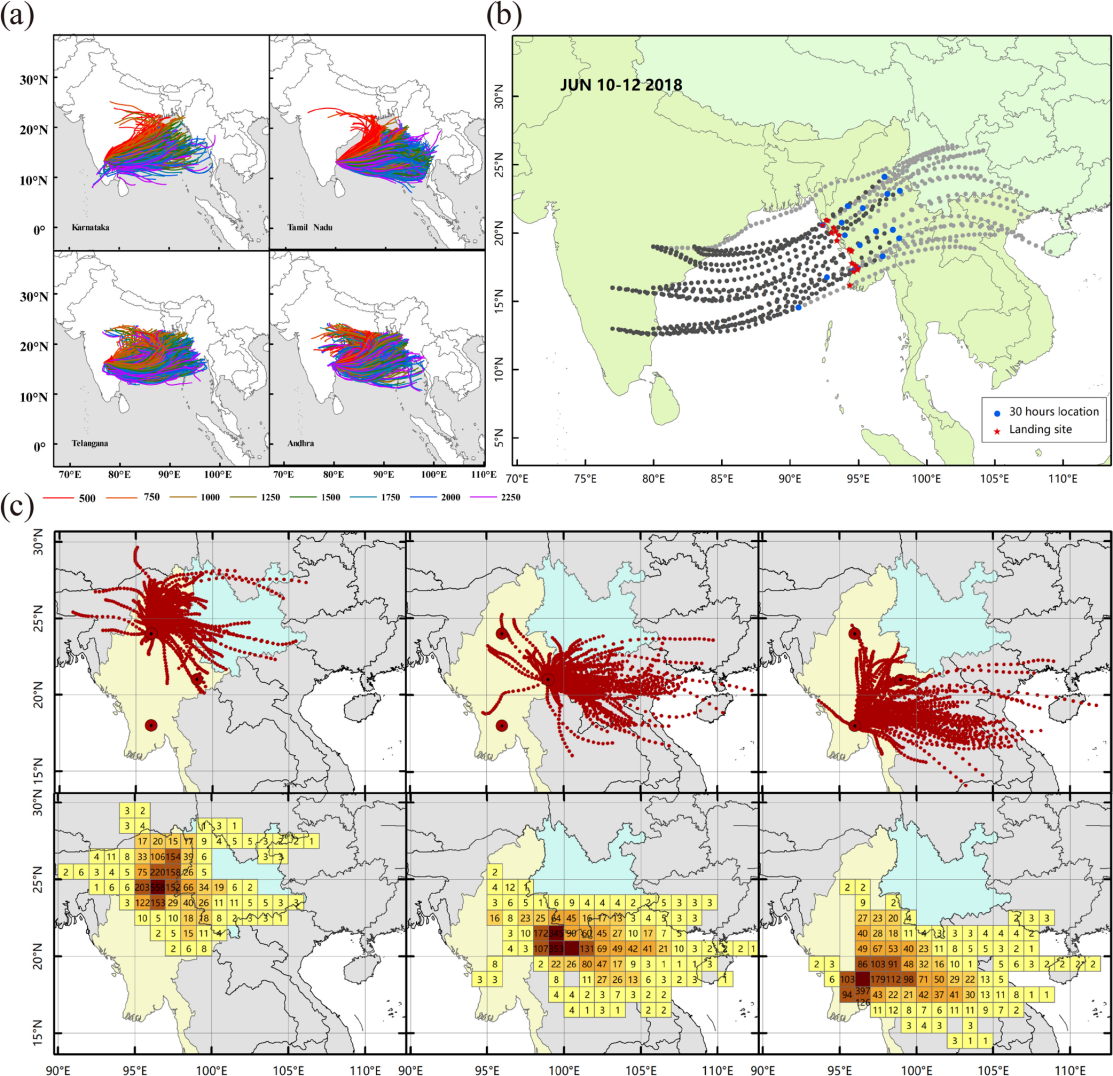
The simulated trajectories of the trans‐sea migration of fall armyworm from India to the Indo‐China Peninsula. (a) The simulated trajectories of the trans‐sea migration of fall armyworm from India to the Indo‐China Peninsula in 2017 and 2018. (b) Migration trajectories of fall armyworm from India to Myanmar, from 10 to 12 June 2018. (c) Migration trajectories and frequency of landing points of fall armyworm in northern, eastern, and southern Myanmar from June to August 2018.

We conducted a trajectory simulation analysis for four regions in southern India where fall armyworm was reported earlier. We found that some migratory tracks reached the Indo –China Peninsula in each of these regions (Figure 5a). On this basis, the spatiotemporal range of the trajectory simulation for India was further expanded, which identified some very typical migration trajectories. For example, in 10–12 June 2018, all the tracks from the six simulation sites reached Myanmar. The average flight duration to landing was 27.6 h, while the shortest was 22 h (Figure 5b).

After reaching India, the fall armyworm began to colonize and spread across East Asia. The simulated trajectories showed that the migration distance of the fall armyworm was shortened. Statistics on the landing points also support this view. Therefore, after settling in Myanmar, the fall armyworm needed to complete at least one generation migration before they could invade China (Figure 5c). In addition, from the trajectories' distribution of different regions in Myanmar, we suppose that eastern Myanmar is the direct source area of fall armyworm that first appeared in China.

### 3.7 Summary of Migration from the Americas to the Indo‐China Peninsula

**FIGURE 6:**
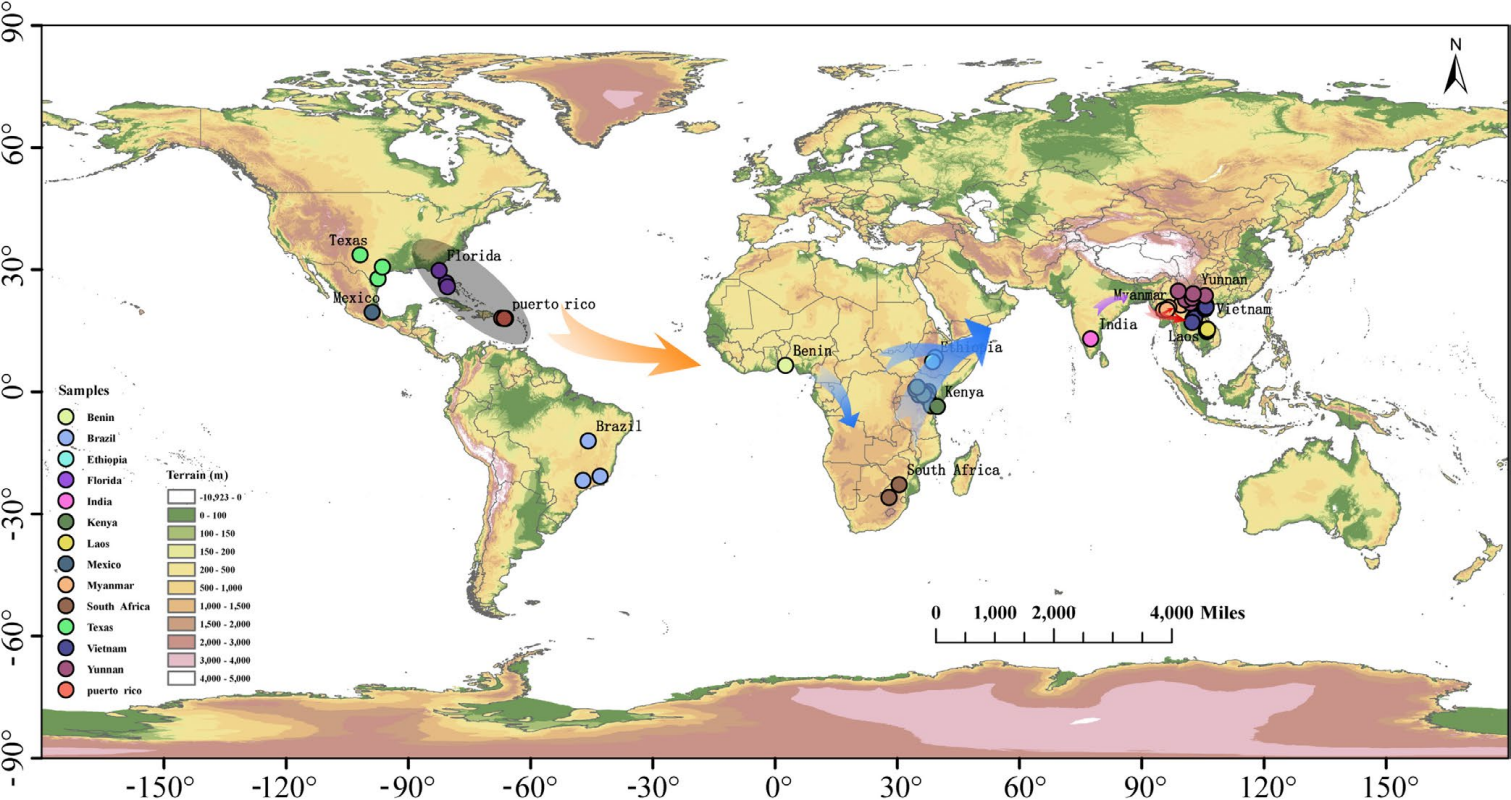
The global migration of the fall armyworm. The arrows in the figure represent the direction of migration and diffusion.

The first migration event was identified as that from America to Africa, with W1 populations identified as potential invasive populations in Africa. The low possibility of trans‐sea migration between the Americas and Africa also suggests that fall armyworms may have spread from the Americas to Africa through trade (Figure S1 and Figures 3 and 6). The second migration event was that from Africa to India. The Indian population was identified as a heterozygous population derived from the E1 population (Figures 4 and 6). The third migration event was that from India to the Indo‐China Peninsula. The fall armyworm reached the Indo‐China Peninsula by means of natural trans‐sea migration from India and then invaded Yunnan after breeding for one generation in Myanmar (Figures 5 and 6). This result supports the views of “spread of African origin” and “spread of Myanmar populations into China”.

## 4 Discussion

In this study, we provided an in‐depth insight into the invasion of the fall armyworm in the Eastern Hemisphere based on genome‐wide data from 124 fall armyworm individuals and trajectory simulation. A low likelihood of trans‐sea migration between the Americas and Africa indicated that fall armyworms may have spread through trade. The results of the integrated individual assignment tests and migration path simulations suggested that the W1 (Florida, Texas, and Puerto Rico) population may be the possible source of the invasive populations in Africa. Our findings also demonstrated that the Indian population was heterozygous, derived from the E1 (Benin, Ethiopia, and South Africa) population, which was followed by invading Yunnan from Myanmar and the Indo‐China Peninsula by natural trans‐sea migration.

In recent years, the fall armyworm has spread widely in the entire Eastern Hemisphere since its invasion of the African continent, posing an unprecedented challenge to the biosecurity of various countries. At present, the invasion and origin of the fall armyworm are of high research interest. Three significant cross‐border migrations were necessary for the fall armyworm to move from the Americas to China. The first was the American –African migration. The current view is that the Benin population in Africa was the “bridgehead” of the first invasion, and the first discovery of fall armyworm in West Africa supports this view (Goergen et al. 2016). Our phylogenetic tree and population structure results support this conclusion by showing that the Benin population is genetically closely related to the American population (Figure 1c). However, the view that Africa had one or more origin groups is still debated by researchers (Zhang et al. 2023). To determine the origin of the African fall armyworm, we considered the American population as the source group and the African population as the distribution group. The results showed that all the African populations were assigned to the W1 population (Figure 3a). In addition, simulations of wind patterns and migration trajectories between the Americas and Africa supported the spread of the fall armyworm to Africa via freight or transportation, with the likelihood of natural transoceanic migration between these continents being less plausible (Figure S1 and Figure 3b) (Cock et al. 2017). In addition, previous research reported a single panmictic population within the Americas (Schlum et al. 2021). Our study revealed the existence of at least three distinct clusters in the Western Hemisphere. A Mexico population from Central America isolated in a single branch, which appears to have independent population characteristics, indicative of earlier geographical origins (Figure 1a,c).

The migration from India to the Indo‐China Peninsula is the third cross‐border migration of the fall armyworm from the Americas to China. Rane et al. conducted a study on mitochondrial genomes and 870 SNPs, finding significant genetic differentiation between the fall armyworm populations in Yunnan, China, and those in Myanmar, Laos, and Vietnam. Moreover, the FIS values (inbreeding coefficients) of the Eastern Hemisphere populations were consistently negative, which contradicts this study's findings where the FIS values of the Eastern Hemisphere populations were mostly positive with less genetic differentiation among populations. This discrepancy is largely attributed to the different numbers of SNP loci involved in the genetic evolutionary analysis and the different populations included in the FIS value calculations (Rane et al. 2023). Evolution of the fall armyworm in the Eastern Hemisphere has been difficult to unravel, which is mainly due to the short invasion event and frequent gene exchange of the invasive population. Our previous study showed that the Yunnan population is closely related to the African populations (Gui et al. 2022). However, the relationship among the Southeast Asia populations including Yunnan and India has not been explored. In the present investigation, we added resequencing data from India and Southeast Asian countries, focusing on the migration history of the fall armyworm from Africa to China. The results of genetic diversity and assignment tests indicated that the E3 population is closely related to the E1 population (Table 1 and Figure 4a). The FST values of E1–E3, E3–E4, and E4–E5 decreased in sequence, which is consistent with the timeline of the fall armyworm's discovery from Africa to China (Figure 2b). Population genetics provides genetic evidence for the spread of the fall armyworm from Africa to India and subsequently to the Indo‐China Peninsula and China, indicating its genetic homogeneity from Africa to China. In addition, the trajectory simulation also supports the possibility of migration from Africa to China (Figures 4b and 5).

The complex invasion history and the limitations of microsatellite markers require more genetic markers to support the analysis of genetic evolutionary relationships of the fall armyworm (Nagoshi et al. 2020; Nayyar et al. 2021; Withers et al. 2021). Therefore, we do not recommend utilizing COI genes or mitochondrial genomes for population structure analysis to infer the invasion history of the fall armyworm. In this study, we inferred the population structure of the fall armyworm using whole genome sequences from 124 individuals collected from 14 geographical locations, covering most of the distribution range from the Americas to China, and the high resolution of whole genome sequences somewhat compensates for the impact of time and location on accurately exploring the genetic evolutionary relationships among different fall armyworm populations (Figure 6 and Table S1). In addition, we also verified the results by means of trajectory simulation. Like previous studies, our study still supports the prevailing view of the “spread of African origin” (Nagoshi et al. 2018, 2020; Schlum et al. 2021; Yainna et al. 2022).

## 5 Conclusions

We provide a thorough understanding of the fall armyworm invasion from the Americas to the Indo‐China Peninsula using trajectory simulation and genome‐wide data from fall armyworm individuals gathered from 14 locations across three continents.

## Conflicts of Interest

The authors declare no conflicts of interest.

## Supporting information


Figure S1

Table S1

Table S2

Table S3

Table S4

Table S5

Table S6

## Data Availability

The SNP dataset that supports the findings of this study has been deposited in the European Nucleotide Archive with the primary accession code PRJEB73569.
