# Supplementary figures and images for: Population Genetics and Trajectory Simulation Reveals the Invasion Process of the Fall Armyworm (Spodoptera frugiperda) in the Eastern Hemisphere
Source: Evol Appl. 2025 Feb 24;18(2):e70086. doi: 10.1111/eva.70086 (PMC11848417; doi:10.1111/eva.70086)

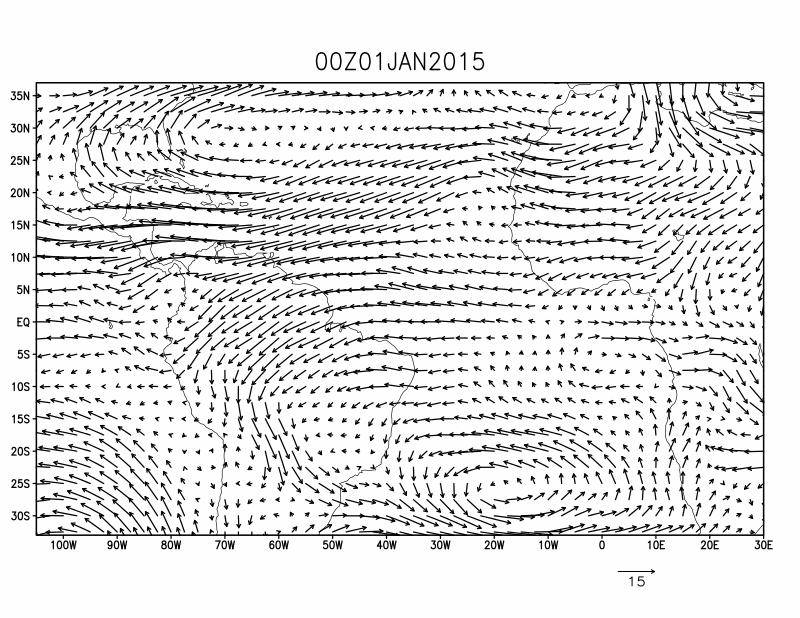

Supplement: Supplementary file 1 — Figure S1 [file EVA-18-e70086-s003.gif]
